# Efficacy of Whey Protein Film Incorporated with Portuguese Green Tea (*Camellia sinensis* L.) Extract for the Preservation of Latin-Style Fresh Cheese

**DOI:** 10.3390/foods11081158

**Published:** 2022-04-16

**Authors:** João Robalo, Maria Lopes, Olga Cardoso, Ana Sanches Silva, Fernando Ramos

**Affiliations:** 1 Faculty of Pharmacy, University of Coimbra, 3000-548 Coimbra, Portugal; joao.dade.robalo@gmail.com (J.R.); mlopes108@gmail.com (M.L.); ocardoso@ci.uc.pt (O.C.); framos@ff.uc.pt (F.R.); 2 Chemical Process Engineering and Forest Products Research Centre, 3030-194 Coimbra, Portugal; 3 National Institute for Agrarian and Veterinarian Research (INIAV), I.P., 4485-655 Vairão, Portugal; 4 Center for the Study of Animal Science (CECA)—Institute of Sciences, Technologies and Agroenvironment (ICETA), University of Porto, 4099-002 Porto, Portugal; 5 Associated Laboratory for Green Chemistry (LAQV) of the Network of Chemistry and Technology (REQUIMTE), University of Porto, 4099-002 Porto, Portugal

**Keywords:** active packaging, antimicrobial activity, antioxidant capacity, edible film, fresh cheese, green tea, whey protein

## Abstract

Fresh cheese composition favors the growth of microorganisms and lipid oxidation, leading to a short shelf life. Whey protein concentrates can be used to produce active films in which green tea (*Camellia sinensis* L.) extract, rich in bioactive compounds, namely catechins, can be incorporated. Thus, the main objective of this study was to evaluate the efficacy of an edible active film, incorporated with green tea extract, to preserve goat and mixture (goat and sheep) fresh cheeses. Our results demonstrated that Portuguese green teas (antioxidant activity coefficient—AAC = 746.7) had superior antioxidant capacity to that of the evaluated Asian green tea (AAC = 650). Furthermore, green tea produced from the leaves of the new Portuguese Chá Camélia tea plantation had the highest potential to retain the antioxidant capacity (97.3%). Additionally, solid–liquid extractions led to extracts with higher antioxidant activity (AAC = 1500), but Soxhlet extractions presented higher yield (43%). Furthermore, the active film incorporated with Portuguese green tea extract exhibited a high antioxidant capacity (AAC ≈ 595.4). In addition, the active film effectively delayed the lipid oxidation of the evaluated fresh cheeses (3.2 mg MDA Eq/kg) when compared with the control (4.2 mg MDA Eq/kg). Moreover, the active films effectively inhibited the growth of microorganisms, especially *E. coli* (1.5 × 10 CFU/g), when compared with the blank (2.2 × 10^2^ CFU/g). This study suggests that the new whey protein film incorporated with Portuguese green tea extract has the potential to be used to extend fresh cheese shelf life.

## 1. Introduction

Polyethylene, polypropylene, and polyamide are the most common polymers used for cheese packaging; however, legislation concerning the migration of chemicals from food-contact materials into cheese can restrict the additives used in their formulations. In addition, the characteristics of these polymers, such as nonbiodegradability and nonedibility, can lead to environmental issues. These significant problems have motivated the research community to search for new packaging solutions for cheese [1]. The cheese industry faces yet another challenge, as fresh cheeses (classified as unripened cheeses) are a very perishable food product. According to the samples used in this research, when stored properly, in refrigerated temperatures (0–7 °C) in an unopened package, fresh cheese can last between 8 and 12 days. However, after opening the package, fresh cheese is acceptable for consumption for 1–2 days. Because of their high pH (>5) and water activity, fresh cheeses are particularly vulnerable to postprocess microbial contamination [2,3,4,5]. During handling and storage, fresh cheese is vulnerable to the growth of nonpathogenic and pathogenic microorganisms. The growth of bacteria, yeast, and molds may not only reduce their quality but affect their safety, since colonization by pathogenic bacteria, such as *Escherichia coli*, *Salmonella*, *Staphylococcus aureus*, and *Listeria monocytogenes*, may endanger consumers by causing foodborne diseases [1,6,7]. In addition, lipid oxidation can alter the type and concentration of certain chemical compounds in cheese, altering the cheese’s nutritional value and causing the loss of liposoluble vitamins and organoleptic properties, resulting in unpleasant tastes and/or aromas and shortening the shelf life (i.e., the length of time during which the food maintains its acceptable or desirable characteristics under specified storage and handling) of the food [8].

Active edible films are edible polymeric materials incorporated with biologically active agents, such as antioxidants or antimicrobials, able to reduce the risk of foodborne bacteria growth and fungal contamination or inhibit oxidation, allowing improvement of packaged foods’ shelf life and quality [9,10,11]. Whey protein is a nutrient-rich by-product of the cheese industry that can be processed into whey protein concentrate (WPC) powders, which can be used as film-forming biopolymers in edible packaging [1,12]. Whey protein has already been applied to edible coatings and film formulations developed for unripened cheeses [13,14,15,16]. Whey protein-based edible films have exhibited not only good mechanical properties, compared with those of standard synthetic polymer films, but good barrier properties against aromas, lipids, and oxygen. However, their hydrophilic nature limits their capacity to act as barriers against water vapor [17,18]. Plasticizers (e.g., glycerol—E 422) can be added to surpass this limitation. This way, whey protein-based films obtain better moisture resistance and improve their resilience (extensibility and flexibility) [17]. Additionally, whey proteins can be obtained from renewable resources and degrade faster than other polymeric materials [10]. To be effective, a whey protein-based edible film requires, besides a polymeric matrix, an active agent. One of the latest active packaging trends is using natural plant extracts as antioxidant and antimicrobial agents [13,19]. Within natural plants extracts, green tea (*Camellia sinensis* L.) extract, because of its physicochemical composition, is a promising antioxidant and antimicrobial agent to be incorporated in edible packaging intended to potentially extend fresh cheese shelf life.

Green tea contains, predominantly, caffeine, a known central nervous system stimulant; vitamins; carbohydrates; amino acids; and polyphenols, such as flavonoids, anthocyanins, and phenolic acids [20,21,22]. Polyphenols are phytochemicals that have drawn increasing scientific attention because of their potential therapeutic effects against a wide variety of diseases [23]. Notably, during the green tea manufacturing process, polyphenol oxidase activity is inactivated, preventing any oxidation reaction from occurring. Therefore, when compared with other types of teas, green tea contains much higher amounts of phenolic compounds, since it maintains not only its original structure but its overall composition [21,24]. Composed essentially by polyphenols, mainly catechins (including epicatechin, epigallocatechin, epigallocatechin-gallate (EGCG), and epicatechin-gallate), and caffeine (2.5–4% dry weight), green tea extracts may be natural antioxidant agents [25,26,27]. Moreover, green tea extracts and EGCG have been shown to have the capacity to suppress foodborne pathogens such as *Staphylococcus aureus*, *Escherichia coli*, *Listeria monocytogenes*, *Salmonella typhimurium*, *Clostridium perfringens*, *Pseudomonas aeruginosa*, *Pseudomonas fragi*, *Helicobacter pylori*, and *Brochothrix thermosphacta* and therefore may be natural antimicrobial agents as well [6]. This actively demonstrates that green tea extracts may act as antioxidant and antimicrobial agents when incorporated into biopolymeric matrices such as whey. For instance, Castro et al. [17] and Andrade et al. (2019) [8] developed whey protein-based films incorporated with green tea extracts and applied them to salmon and salami, respectively, to inhibit lipid oxidation. Portugal has, to date, three tea plantations, Gorreana, Chá Camélia, and Chá Porto Formoso, among which Gorreana and Chá Camélia produce outstanding green teas that can be used to obtain green tea extracts. Gorreana currently has the largest tea plantation in Europe, and it is internationally recognized not only for the quality and uniqueness of its teas (black, green, and oolong) but also for the production of 100% organic, chemical-free teas, as the use of synthetic chemical pesticides is not justified given that the island’s climate does not allow the development of tea plant pests. Gorreana produces and markets three varieties of green tea, namely “Encosta de Bruma”, “Pérola”, and “Hysson”. The latter variety was selected as a sample in this study. The Chá Camélia plantation was born on the northern Portuguese coast and is the only producer of artisanal, organic, Asian-style green tea in mainland Portugal; this tea was used as a sample in this study. In addition, it produces “Florchá”, which consists of dehydrated flowers of *Camellia sinensis* L. that are used to make delicate, caffeine-free herbal infusions, which were also used as a sample in this study. Finally, the Porto Formoso tea factory, located on the north coast of the island of São Miguel, currently produces black tea of the following varieties: “Orange Pekoe”, “Pekoe”, “Broken Leaf”, and “Azores Home Blend”. However, none of these varieties were selected for this study.

A wide variety of edible films and coatings have been developed for multiple types of cheese [1]. However, according to the bibliography, only six edible packages have been developed to date. In addition, even though multiple polymeric matrices (cassava starch [28], liquid whey protein concentrate [13], chitosan [29], chitosan plus whey protein [15], and furcellaran plus whey protein isolate [14,16]) and active compounds (yerba mate and white tea [14], green tea and Pu-erh [16], *Lactobacillus acidophilus* [28], Chinese cinnamon bark [13], and bacteriocins [29]) have been used to develop active packages for several unripened types of cheese (fresh soft rennet-curd cheese [14], quark [16], Manaba fresh white cheese [28], ricotta cheese [15], Eastern European curd cheese [13], and Colombian fresh cheese [29]), no scientific findings are yet available on edible films’ application to and effect on Latin-style fresh cheese, a type of fresh cheese produced by enzymatic coagulation of milk with rennet, without adding starter cultures, traditionally made in the Iberian peninsula from pasteurized goat or cow milk. Latin-style fresh cheeses do not require the addition of starter cultures and therefore present high pH values (>5.0). In addition to having low salt and high moisture content, they are usually made without preservatives. These characteristics favor the growth of microorganisms and reduce the cheeses’ shelf life [3,7]. Therefore, it is interesting to evaluate the effect of a whey protein film incorporated with green tea extract on the preservation of this type of fresh cheese, as it is a nonripened cheese widely consumed in the Iberian peninsula (particularly in Portugal) and very perishable, demanding consumption shortly after production.

In this context, the present research study’s main objective was to characterize and compare the antioxidant properties of Portuguese (Gorreana and Chá Camélia) and Asian (Happy Flora) green teas, including Chá Camélia green tea from 2020 production; optimize the preparation of green tea extract; and evaluate the efficacy of an edible active film incorporated with green tea extract in preserving goat and mixed (goat and sheep) fresh cheeses. This packaging incorporated Portuguese green tea extract to potentially extend Latin-style fresh cheese’s shelf life. In addition, this was the first study evaluating the antioxidant capacity of Chá Camélia, the first green tea from continental Portugal.

## 2. Materials and Methods

### 2.1. Green Tea Samples

In this study, three Portuguese samples from *C. sinensis* L. were used, a green tea sample from the oldest tea plantation in Europe, called Gorreana (37°49′06.7′′ N 25°24′09.1′′ W), and two samples from the first tea plantation in continental Portugal, Chá Camélia (41°19′56.8′′ N 8°38′31.2′′ W). The latter samples were produced according to traditional organic farming methods. From the Gorreana plantation, located on the island of São Miguel (Azores), the “Hysson” variety was chosen. This tea is produced after harvesting, in July and August, from the first three leaves of *C. sinensis*. From the Chá Camélia plantation, located on the northern Portuguese coast, two samples were evaluated. One was obtained from the leaves of *C. sinensis* from the first green tea harvest, Asian style, in continental Portugal, and the other was produced in autumn, resulting from the dehydration of flowers of the tea plant (*C. sinensis*). Furthermore, an Asian tea marketed by ADP, LDA (39°26′55.2″ N 8°47′20.4″ W) as Happy Flora was selected. The Gorreana-brand and Happy Flora brand green teas were purchased locally in a commercial area in Coimbra. The samples from the Chá Camélia brand (green tea and *C. sinensis* flowers) were kindly supplied by the company.

### 2.2. Preparation of Green Tea Infusions

The teas were prepared as traditionally as possible. Two hundred milliliters of tap water was heated to 75 °C, and immediately afterward, 2 g of *C. sinensis* leaves/flowers, weighed previously, was soaked for 3 min, with the aid of a tea filter, in the absence of a sachet. At this stage, for each variety, 4 infusions were prepared. The 1st infusions were prepared using dry leaves of different varieties of green tea, which were designated as G (Gorreana), H (Happy Flora), CA (Chá Camélia—leaves), and CB (Chá Camélia—flowers). Then, the 2nd, 3rd, and 4th infusions were prepared sequentially from the reuse of the same leaves/flowers, these being designated by *2, *3, and *4, respectively (* corresponds to G/H/CB/CA). Finally, the antioxidant capacity, total phenolic content (TPC), and total flavonoid content (TFC) of the infusions and teas were evaluated. The retention potential of the samples was evaluated by comparing the antioxidant properties, TPC, and TFC of the 4th infusion with those of the 1st infusion of the same sample.

### 2.3. Extraction of Green Tea Extracts

#### 2.3.1. Conventional Solid–Liquid Extraction

Initially, the *C. sinensis* samples were ground, homogenized, and sieved. Five grams of each sample (AS 220.R1 PLUS scale) was subjected to solid–liquid extraction with 50 mL of absolute ethanol (99.8%) and were placed in a horizontal shaker (Edmund Bühler KL2) for 30 min at 400 rpm, followed by centrifugation (Sigma 3–16 K centrifuge, Osterode, Germany) at 5000× *g* for 15 min at 15 °C. Afterward, the supernatant was removed into a pyriform flask, and using a rotary evaporator, the ethanol was evaporated entirely at 40 °C. Next, the extract was removed from the flask using a spatula and stored in a closed container at 5 °C. Then, a solution was prepared using 10 mg of the extract and 10 mL of ethanol. Finally, the antioxidant capacity was evaluated. The yield of the solid–liquid extraction was calculated using the following equation:

(1) $$Yield\;\left(\%\right)=\frac{A-B}{C}\times 100.$$

where *A* represents the weight of the extraction flask with extract, *B* represents the weight of the empty extraction flask, and *C* represents the amount of tea sample used to perform the extraction.

#### 2.3.2. Solid–Liquid Extraction with Soxhlet Apparatus

Five grams of each sample was weighed into a Soxhlet cartridge, which was placed in a Soxhlet extractor using 150 mL of absolute ethanol (99.8%) as solvent for 6 h. Using a rotary evaporator, the solvent was completely evaporated at 40 °C. Next, the dry extract was removed from the distillation flask using a spatula and stored in a closed container at 5 °C. Afterward, a solution was prepared using 10 mg of the dry extract and 10 mL of ethanol to allow the evaluation of the antioxidant capacity. The yield of the solid–liquid extraction via Soxhlet apparatus was calculated using Equation (1) as previously described.

### 2.4. Preparation of Whey-Based Protein Films

The method of preparing whey protein films was adapted from Ribeiro-Santos et al. [30]. Initially, ultrapure water was added to the whey protein concentrate (acquired online from Myprotein^®^—“impact whey protein”). Then, homogenization was carried out using a magnetic stirrer, obtaining a solution (8%, *w*/*w* protein). The pH was, when necessary, adjusted to 7.0 using 1M NaOH. Subsequently, the protein was denatured by heating the solution in a thermostatic bath for 30 min at 80 °C. The solution was rapidly cooled in an ice bath to room temperature (+/−23 °C). Glycerol 1:1 (protein–glycerol) was added and mixed into the solution. Furthermore, 2.5% (*w*/*w*) of the extract was incorporated into the formulation and then homogenized using an Ultra-Turrax at 14,000 rpm for 2 min. The solution was distributed in Petri dishes (13.5 mL/100 cm^2^). Finally, the Petri dishes were incubated in an oven (Memmert 854, Schwabach, Germany) at 40 °C for 24 h. A film without incorporation of extract in the film-forming solution was used as a blank (control). The films were stored in the Petri dishes at refrigerated temperatures. To enable the antioxidant evaluation of the films, a migration assay was performed; 6 cm^2^ of each film (blank and active) was immersed into 10 mL of ethanol (95%) and incubated at 40 °C for 10 days. After the incubation, the antioxidant capacity was immediately evaluated.

### 2.5. Evaluation of the Antioxidant Properties

Before evaluation of antioxidant properties, green tea extract (dry powder format) was diluted in ethanol.

#### 2.5.1. β-Carotene Bleaching Assay

The β-carotene bleaching test allows evaluating antioxidant effects against lipid peroxidation [31]. Specifically, when linoleic acid is in the presence of reactive oxygen species (ROS) or oxygen, a peroxyl radical is formed (LOO•). This radical forms a stable β-carotene radical by reacting with β-carotene, reducing the amount of β-carotene. However, in the presence of an antioxidant, a competitive reaction occurs between the antioxidant and β-carotene with the peroxyl radical [32]. This reaction occurs in an aqueous emulsion of linoleic acid prepared using a phase stabilizer, namely Tween^®^ 40 (Sigma-Aldrich; Madrid, Spain) [33]. Under standard settings, thermal induction (50 °C) results in the oxidation of the fatty acid-generating radicals, which causes the discoloration of the yellow-colored emulsion [34,35]. However, in the presence of an antioxidant, this discoloration can be delayed via the previously mentioned competitive reaction. These antioxidant effects can be quantified through spectrophotometry (470 nm) by measuring the rate at which β-carotene absorbance decays [32,34].

The procedure of this assay followed the method described by Miller and adapted by Andrade et al. [36]. Initially, a solution was prepared by dissolving β-carotene in chloroform (0.2 mg/mL). Next, an emulsion of β-carotene and linoleic acid was prepared using 20 mg of linoleic acid, 200 mg of Tween^®^ 40, and 1 mL of the previously prepared solution. Chloroform was evaporated at 40 °C on a rotary evaporator. Thereafter, 50 mL of ultrapure water was added, and the solution was vigorously stirred. After it was prepared, 5 mL of the emulsion was added to 200 µL of the sample. The samples were kept in a Gerhardt SV24 thermostatic bath (1500 w) at 50 °C for 120 min. Using a Hitachi U-3900 spectrophotometer (Hitachi, Tokyo, Japan), the absorbances of the samples were measured at 470 nm. The antioxidant activity coefficient (AAC) was calculated using Equation (2):

(2) $$AAC=\frac{AS-AC2}{AC0-AC2}\times 1000.$$

where *AS* represents the absorbance of the samples, *AC*0 represents the absorbance of the control before heating, and *AC*2 represents the absorbance of the control after heating.

#### 2.5.2. DPPH Radical-Scavenging Method

The DPPH radical (2,2-diphenyl-1-picryl-hydrazyl) assay is an easy and quick method that allows evaluating a sample’s capability to inhibit lipid oxidation by determining its free radical-scavenging capacity [37,38,39]. In short, the antioxidant reacts with the radical DPPH•, reducing it to diphenylpicrylhydrazine, leading to the discoloration of the solution [33].

Briefly, 2 mL of a methanolic solution of DPPH (14.6 μg/mL) was added to 50 μL of the sample. After homogenization, the solutions were protected from light for 30 min. Absorbance was measured at 515 nm using a Hitachi U-3900 spectrophotometer(Tokyo, Japan. DPPH• inhibition percentage (IP%) was calculated using Equation (3):

(3) $$IP\;\left(\%\right)=\frac{AC-AS}{AC}\times 100.$$

where *AC* represents the absorbance of the control and *AS* represents the absorbance of the sample. The method applied followed the method described by Blois (1958) [40] with some adaptations. A calibration curve (y = 0.6051x + 7.0233, r^2^ = 0.9979) was drawn up by plotting different concentrations of Trolox (5–150 µg/mL) in order to express the results in Trolox equivalent (TE). Results were expressed as µg Trolox equivalent/g or mL of sample.

#### 2.5.3. Determination of Total Flavonoid Content (TFC)

The spectrophotometric assay, based on the production of aluminum complexes and their spectrophotometric determination, is one of the most widely used procedures for determining total flavonoid content [41]. According to the basic concept of the aluminum chloride (AlCL_3_) colorimetric method, AlCl_3_ forms acid-stable complexes with the C-4 keto group and either the C-3 or C-5 hydroxyl group of flavones and flavonols, resulting in a yellow color [42,43,44].

To determine the TFC, the method described by Yoo et al. [45] was applied. Briefly, 4 mL of ultrapure water was added to 1 mL of sample. Then, 300 µL of an aqueous solution of sodium nitrite (5%, *w*/*v*) and the mixture were homogenized. After 5 min, 600 µL of an aqueous aluminum chloride solution (10%, *w*/*v*) was added, and the solution was homogenized again. After 6 min, 2 mL of an aqueous solution of sodium hydroxide (1 M, *w*/*v*) and 2.1 mL of ultrapure water were added. All steps occurred at room temperature. Finally, the samples were homogenized, and the absorbance was measured at 510 nm using a Hitachi U-3900 Spectrophotometer (Tokyo, Japan). A calibration curve (y = 0.0027x − 0.0017, r^2^ = 0.9972) was built for the TFC assay by plotting different concentrations of epicatechin (5–200 µg/mL) in order to express the results in epicatechin equivalent (ET). Results were expressed in mg epicatechin equivalent/g or mL of sample.

#### 2.5.4. Determination of Total Phenolic Compounds (TPC)

The Folin–Ciocâlteu reagent (FCR) assay is commonly used to determine total phenolic content (TPC). This colorimetric assay of phenolic and polyphenolic antioxidants is conducted with Folin’s phenol reagent, a combination of phosphomolybdate and phosphotungstate. The FCR assay is based on the single electron transfer (SET) from phenolic chemicals to FCR in an alkaline solution resulting in a blue-colored chromophore that can be detected spectrophotometrically at 750–765 nm. Specifically, phenolics are energetically oxidized, yielding O_2_, which combines with molybdate to yield colored molybdenum ions, MO^4+^ [33,46].

The content of TPC was determined by the method described by Singleton and Rossi (1965) [47]. Briefly, 1 mL of each sample was mixed with 7.5 mL of FCR (10%, *v*/*v*). After 5 min, 7.5 mL of a 60 mg/mL (*w*/*v*) aqueous sodium carbonate solution was added. After homogenization, the solutions were kept in the dark for 120 min at room temperature. Finally, absorbance was measured using a Hitachi U-3900 spectrophotometer (Tokyo, Japan at 725 nm. A gallic acid calibration curve (y = 0.0064x + 0.0463, r^2^ = 0.9929) was built by plotting different concentrations of gallic acid (5–150 µg/mL) in order to express the results in gallic acid equivalent (GAE). Results were expressed in mg gallic acid equivalent/g or mL of sample.

### 2.6. Fresh Cheese Samples

**Figure 1:**
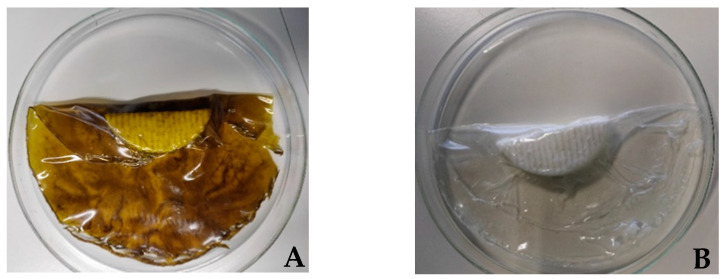
Mixture fresh cheeses packaged with the active film (**A**) and the control film (**B**).

For the microbiological analysis, goat fresh cheese and a mixture fresh cheese (a cheese obtained from a mixture of sheep and goat milk) were acquired at a local cheese factory (Queijaria da Licínia, Rabaçal, Portugal ), while for the lipid oxidation analysis, goat and mixed fresh cheeses were purchased at a commercial store in Coimbra (Portugal). In both situations, after the acquisition or purchase, the fresh cheeses were, on the same day, packaged with both the active and the control films (Figure 1) and stored at 5 °C. The nutritional compositions of the samples can be consulted in Table 1. After a week in storage, microbiological analysis and lipid oxidation evaluations were performed.

### 2.7. Evaluation of the Lipid Oxidation Status (TBARS Assay)

The antioxidant capacity of active packaging, when in contact with two types of fresh cheese (prepared with goat milk and with goat/sheep milk mixture) purchased at a commercial store, was analyzed through the TBARS assay. The two types of cheese were wrapped with the edible active films and with the control films (without green tea extract) and stored at 5 °C to simulate standard storage conditions. After one week of storage at refrigerated temperatures, the assay was carried out. Briefly, 10 mL of trichloroacetic acid (7.5%, *v*/*v*) was mixed with 5 g of each sample and homogenized in a compact stirrer for 1 h at 350 rpm. Then, the samples were filtered through a Whatman number 1 paper filter. Afterward, 5 mL of thiobarbituric acid (2.88 mg/mL, *w*/*v*) (TBA) was mixed with 5 mL of the filtrate. Then, the samples were submitted to 95 °C in a heat block for 30 min and then rapidly cooled in ice for 15 min. The absorbance was measured, using a Hitachi U-3900 spectrophotometer (Tokyo, Japan), at 530 nm against the blank assay (5 mL of water and 5 mL of TBA). For this assay, a calibration curve (y = 17.657x − 0.0326; r^2^ = 0.9905) was built by plotting different concentrations of 1,1,3,3-tetramethoxypropane (10–55 µg/mL) to express the results in mg of malonaldehyde equivalent per kg of sample (mg MDA Eq/kg).

### 2.8. Microbiological Analysis

The antimicrobial capacity of active packages in contact with two types of fresh cheese (prepared with goat milk and with goat/sheep milk mixture) was analyzed by counting the microorganisms present in the cheese. To observe the antimicrobial capacity of the active packages, various parameters were determined: total microorganisms at 30 °C according to ISO 4833-1:2013, total of psychrophiles according to ISO 17410:2019, count of *E. coli* according to ISO 16649-2:2001, and count of coagulase-positive staphylococci according to ISO 6888-1.

The counts of the different microorganisms were carried out on fresh goat cheese and fresh mixture (sheep and goat) cheese produced on the day. The two types of cheese were wrapped with the edible active films and with the control films (without green tea extract) and stored at 5 °C to simulate standard storage conditions. After one week of storage at refrigerated temperatures, microorganism counts were carried out.

### 2.9. Statistical Analysis

For statistical analysis, the GraphPad Prism (v9.1.2) software developed by Dr. Harvey Motulsky (San Diego, California), was used. One-way ANOVAs were performed using a significance level of 0.05. Additionally, Tukey’s multiple comparison tests were performed.

## 3. Results and Discussion

### 3.1. Evaluation of the In Vitro Antioxidant Capacity, TPC, and TFC

In this research study, the antioxidant capacity was evaluated, and the TPC and TFC determined, of tea infusions from different green tea varieties. Subsequently, the same evaluations were performed on different green tea extracts. Finally, the antioxidant capacity was evaluated, and the TPC and TFC determined, of the active edible film.

To assess the antioxidant capacity of the active edible film and green tea infusions and extracts, DPPH free radical-scavenging and β-carotene bleaching methods were selected

To evaluate TPC, a Folin–Ciocâlteu’s reagent assay was used. To evaluate TFC, a spectrophotometric assay based on the production of aluminum complexes and their spectrophotometric determination was used.

#### 3.1.1. Evaluation of the Antioxidant Capacity, TPC, and TFC among Different Green Tea Infusions

This study evaluated the antioxidant capacity of green tea infusions from two Portuguese brands: Gorreana and Chá Camélia. From the Gorreana tea brand, the “Hysson” variety was chosen, and from the Chá Camélia brand, one infusion was prepared from the leaves of *Camellia sinensis* L., and another from the flowers of *C. sinensis*. In addition, the antioxidant capacity of an Asian green tea marketed as Happy Flora was assessed. Furthermore, the potential of retention of the antioxidant capacity was evaluated through antioxidant analysis of infusions made by reusing the same leaves/flowers.

**Figure 2:**
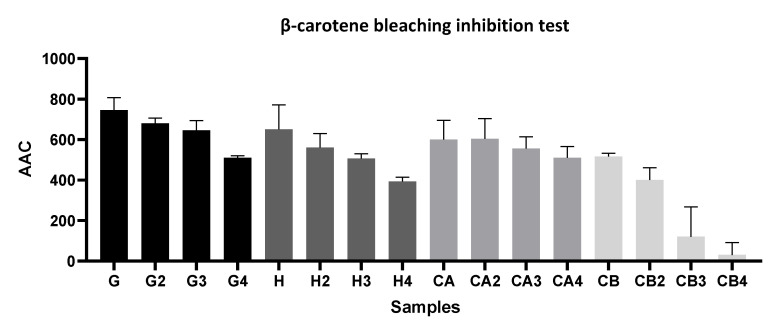
Comparison of the antioxidant activity of different green teas and *C. sinensis* flower infusions. Results of β-carotene bleaching inhibition test are expressed as antioxidant activity coefficients (AAC). G = Gorreana; H = Happy Flora; CA = Chá Camélia (leaves); CB = Chá Camélia (flowers). The numbers 2, 3, and 4 stand for the 2nd, 3rd and 4th infusions obtained by reusing the green tea leaves (G, H, CA) or *C. sinensis* flowers (CB).

**Figure 3:**
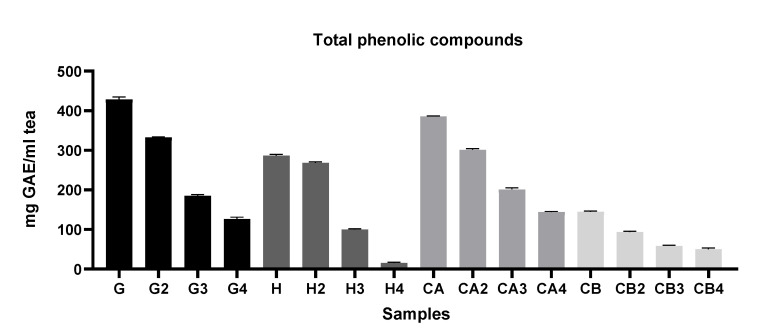
Comparison of the total phenolic compounds of different green teas and *C. sinensis* flower infusions. Results of TPC are expressed in mg of gallic acid equivalent per g of tea (mg GAE/g tea). G = Gorreana; H = Happy Flora; CA = Chá Camélia (leaves); CB = Chá Camélia (flowers). The numbers 2, 3, and 4 stand for the 2nd, 3rd and 4th infusions obtained by reusing the green tea leaves (G, H, CA) or *C. sinensis* flowers (CB).

**Figure 4:**
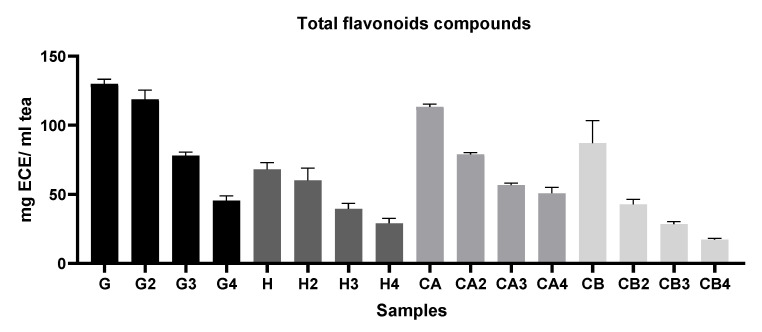
Comparison of the total flavonoid compounds (TFC) of different green teas and *C. sinensis* flowers’ infusion. Results of TFC are expressed as mg of epicatechin equivalent per g of tea (mg ECE/ml tea). G = Gorreana; H = Happy Flora; CA = Chá Camélia (leaves); CB = Chá Camélia (flowers). The numbers 2, 3, and 4 stand for the 2nd, 3rd and 4th infusions obtained by reusing the green tea leaves (G, H, CA) or *C. sinensis* flowers (CB).

**Figure 5:**
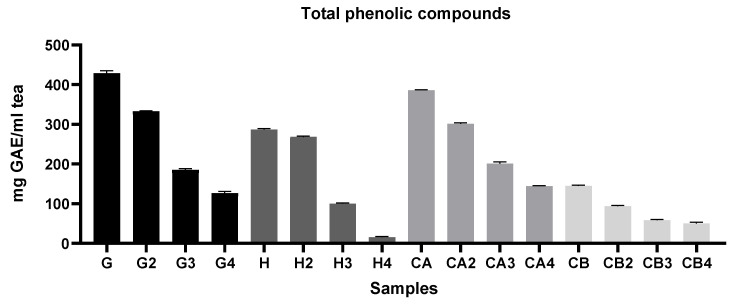
Comparison of the antioxidant activity of different green teas and *C. sinensis* flower infusions. Results of DPPH radical inhibition test are expressed in µg Trolox equivalent per g of tea (µg TE/mL tea). G = Gorreana; H = Happy Flora; CA = Chá Camélia (leaves); CB = Chá Camélia (flowers). The numbers 2, 3, and 4 stand for the 2nd, 3rd and 4th infusions obtained by reusing the green tea leaves (G, H, CA) or *C. sinensis* flowers (CB).

In general Figure 2, Figure 3 and Figure 4 show that infusions from Gorreana green tea had the highest antioxidant activity and that the analyzed infusions of green tea from the Gorreana (G) and Chá Camélia (CA) plantations had superior phenolic and flavonoid content than the analyzed infusion of Asian green tea (H). Furthermore, Figure 2, Figure 3, Figure 4 and Figure 5 demonstrate that, overall, tea from the Chá Camélia plantation, obtained from the leaves of *C. sinensis* (CA), had the highest potential to retain the antioxidant capacity.

Figure 3, Figure 4 and Figure 5 corroborate the previously established assumptions. Figure 2 shows that the Gorreana (G) tea had a greater capacity to inhibit lipid peroxidation (AAC_G_ = 746.7). However, there were no significant differences (*p* > 0.05) among the Gorreana (G), Chá Camélia (AAC_CA_ = 600), and Happy Flora (AAC_H_ = 650) teas.

Additionally, considering the AAC of teas produced from fresh leaves (G, H, CA), *C. sinensis* flower infusions, (CA), and teas/herbal infusions obtained from their reuse (*2,*3,*4), it appears that the green tea obtained from the leaves of the Chá Camélia tea plantation showed the highest capacity to retain its antioxidant activity (85%) compared with the other samples (Gorreana ≈68.3%; Chá Camélia (flowers) ≈5.8%; Happy Flora ≈60.5%). As far as we know, the β-carotene bleaching assay has not been used before to evaluate the capacity of green tea infusions to inhibit lipid peroxidation, and therefore, it is not possible to compare our results to those of other research studies.

Analysis of Figure 5 shows that the green tea obtained from the leaves of the Chá Camélia tea plantation had the highest capacity to capture DPPH● free radicals (≈139.8 µg TE/mL). However, excluding tea/herbal infusions produced through the third reuse of leaves/flowers, there were no significant differences (*p* < 0.05) among tea varieties regarding antioxidant capacity. The infusion of Chá Camélia green tea obtained from leaves had the highest potential to retain antioxidant capacity (≈97.3%) when compared with the other infusions obtained from flowers (≈73%) or leaves Gorreana (≈62.4%) and Happy Flora (≈78.8%).

Several studies have used the DPPH radical scavenging assay to evaluate the antioxidant capacity of green tea infusions. However, past publications reported the results of the DPPH assay in different units, so we were unable to compare those results with those of the current study.

Analysis of Figure 3 indicates significant superiority (*p* < 0.05) of the Portuguese tea samples obtained from leaves in terms of total phenolic compound content, with the Gorreana being considerably (*p* < 0.05) richer in phenolic compounds (≈428.4 mg GAE/mL tea) than the Chá Camélia (≈385.5 mg GAE/mL tea). Considering the first and fourth infusions, the Chá Camélia tea showed the highest potential to retain phenolic compounds (≈37.4%). However, the difference was not as discrepant as for the Gorreana tea (≈30%). It should be noted that, despite presenting the second-highest potential for retention of phenolic compounds (≈34.5%), the infusion obtained from flowers of *C. sinensis* (Chá Camélia), presented much lower values (≈144.9 mg GAE/mL tea) than the two aforementioned green teas. In addition, is important to note that even though the Happy Flora tea had the lowest values, it had the highest capacity to retain phenolic compounds (≈93.6) when considering the first and second infusions.

A review of the literature allowed us to verify that, even though TPC is a widely used parameter for evaluating the antioxidant activity of tea infusions, it is challenging to compare TPC values with those obtained in other studies. Even though the Folin–Ciocâlteu reagent method is one of the main techniques used to determine TPC, many procedures have been applied. For instance, Kodama et al. [48] and Almeida et al. [49] determined the TPC of tea infusions using the Folin–Ciocâlteu reagent method but according to Singleton, Orthofer, and Lamuela-Raventos (1998) [50]. Komes et al. (2010) [51] used the same reagent but determined the TPC according to the modified method of Lachman, Hosnedl, Pivec, and Orsák (1998) [52]. In addition, the units in which the results have been displayed have varied among studies. For instance, Kodama et al., 2010 expressed the results as mg of catechin equivalent/200 mL.

Finally, Figure 4 shows values similar to those in Figure 3, which was expected. Thus, Portuguese teas obtained from the leaves had higher total flavonoid content. However, although the Gorreana tea was richer in flavonoids (≈130 ECE/mL tea), there were no significant differences (*p* > 0.05) from the Chá Camélia tea (≈113.3 mg ECE/mL tea). Once again, the Chá Camélia tea was superior in retention potential (44.9%) to other samples. It should be noted that although Happy Flora had a higher retention potential than the Gorreana tea (approximately 42.8% and 35%, respectively), the total flavonoid content in Happy Flora tea (68 mg ECE/mL tea) was much lower than that in the Gorreana tea. Our infusions presented higher total flavonoid content than the matcha green teas evaluated by Jakubczyk et al. [53]. In addition, the TPC and TFC values of our infusions were higher than those of the infusions evaluated by Komes et al. (2010) [51], among which the green tea blend Rose of the Orient had lower TPC and TFC values (880 and 440 mg/L, GAE, respectively), whereas the bagged Twinings of London green tea (2560 and 1920 mg/L GAE, respectively) and powdered matcha green tea (2230 and 1630 mg/L, respectively) had higher TPC and TFC values

#### 3.1.2. Evaluation of the Antioxidant Capacity of Green Tea Extracts

In the present study, solid–liquid extractions and Soxhlet extractions were performed simultaneously using the teas previously evaluated to compare the antioxidant capacity and yield of green tea extracts. Chá Camélia tea varieties were excluded from this trial because of the limited number of samples available.

**Figure 6:**
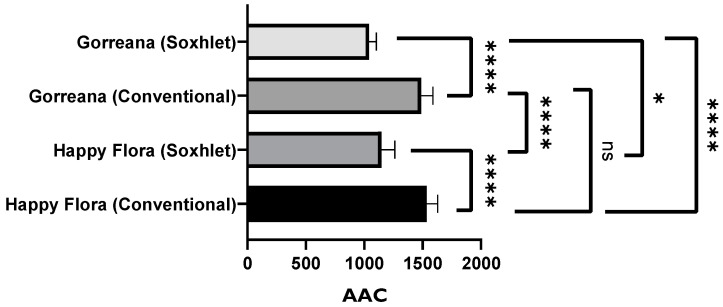
Comparison of green tea extracts prepared by two different extraction methods. β-carotene bleaching inhibition test results are expressed as AAC. **** = *p* ≤ 0.0001,* = *p* ≤ 0.05, ns = *p* > 0.05.

**Figure 7:**
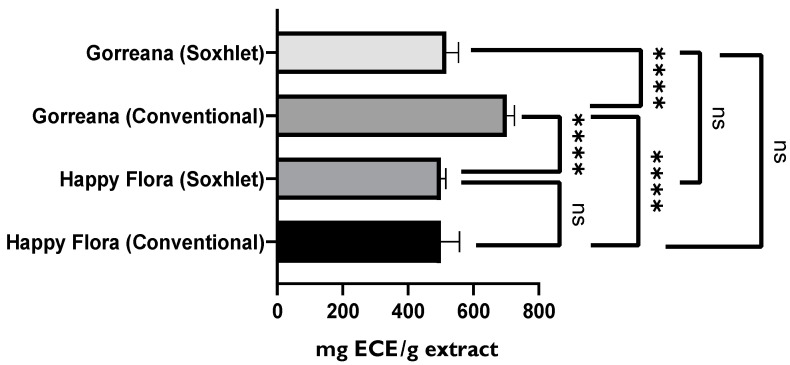
Comparison of green tea extracts prepared by two different extraction methods. Total flavonoid content is expressed in mg of epicatechin equivalent per g of extract (mg ECE/g extract). **** = *p* ≤ 0.0001, ns = *p* > 0.05.

**Figure 8:**
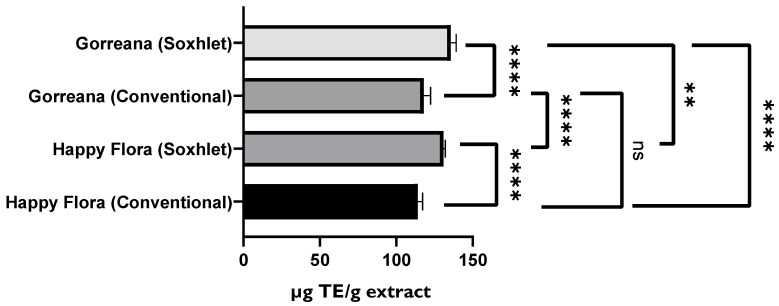
Comparison of green tea extracts prepared by two different extraction methods. Results of DPPH radical inhibition test are expressed as mg Trolox equivalent per g of extract (µg TE/g extract). **** = *p* ≤ 0.0001, ** = *p* ≤ 0.01, ns = *p* > 0.05.

**Figure 9:**
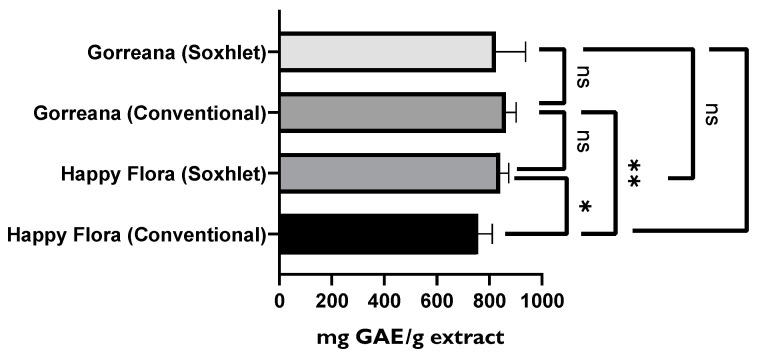
Comparison of green tea extracts prepared by two different extraction methods. Total phenolic compound content is expressed as mg of gallic acid equivalent per g of extract (mg GAE/g extract). ** = *p* ≤ 0.01, * = *p* ≤ 0.05, ns = *p* > 0.05.

In a brief analysis of Figure 6, Figure 7, Figure 8 and Figure 9, it is possible to conclude that the extracts obtained through the method described by Andrade et al. [36] demonstrated superior antioxidant capacity to that of the extracts obtained through solid–liquid extraction with the Soxhlet apparatus. In a more detailed analysis, Figure 6 confirms that the extracts obtained by the conventional solid–liquid extraction method inhibited lipid peroxidation more efficiently, presenting AAC values around 1500. However, there were no substantial differences among the analyzed green tea samples when the same extraction method was applied (*p* > 0.05).

Furthermore, Figure 7 demonstrates that the extract of Gorreana obtained by conventional solid–liquid extraction contained a higher content on total flavonoids (701.5 mg ECE/g extract). However, the results shown in Figure 8 do not support the previously established premise. By analyzing Figure 8, it can be inferred that the extracts obtained through Soxhlet extraction have a greater capacity to capture free radicals DPPH● (Gorreana ≈ 138.2 µg TE/g extract; Happy Flora ≈ 131.2 µg TE/g extract) than extracts obtained through the conventional solid–liquid extraction method (Gorreana ≈ 118 µg E.T./g/ Happy Flora ≈ 114 µg E.T./g), with no significant differences among green tea extracts when applied the same extraction method (*p* > 0.05).

Furthermore, regarding total phenolic compound content, there were no significant differences (*p* > 0.05) between Gorreana extracts, even when different extraction methods were applied. However, as shown in Figure 9, significant differences (*p* < 0.05) between the Happy Flora extracts were observed. Furthermore, there were considerable differences between the Happy Flora extract obtained through conventional solid–liquid extraction (≈758.3 mg GAE/g extract) and the Gorreana extract obtained through the same method (≈863.1 mg GAE/g extract). In addition, there were considerable differences between the previously mentioned Happy Flora extract and the Gorreana extract obtained by Soxhlet extraction (≈833.7 mg GAE/g extract).

Overall, the antioxidant capacity of our extracts was higher than that of the extracts from a study by Martins et al. (2018) [54]. All of our extracts presented higher AAC except Gorreana extracts obtained through Soxhlet extraction. According to our results, the Happy Flora extract (conventional solid–liquid extraction) exhibited an AAC of 1536, the Gorreana extract exhibited an AAC of 1488, and the Happy Flora extract (Soxhlet) exhibited an AAC of 1210, while according to the results of Martins et al., extracts from commercial green tea, green tea from capsules, “Hysson” green tea, and “Encosta da Bruma” green tea exhibited AACs of 918, 1079, 1144, and 1178, respectively. Only the Gorreana extracts obtained through Soxhlet extraction exhibited a lower, but still high, AAC (1051) than the green tea extracts from the previously mentioned study. In addition, our DPPH results were very high. Extracts from Gorreana (conventional), Gorreana (Soxhlet), Happy Flora (conventional), and Happy Flora (Soxhlet) exhibited Trolox equivalent antioxidant capacities (TEAC values expressed in µg TE/g extract) of 117.9, 138.2, 114, and 131.2, respectively. According to Martins et al., extracts from commercial green tea, green tea from capsules, “Hysson” green tea, and “Encosta da Bruma” green tea exhibited TEACs (mg TE/g extract) of 0.917 ± 0.09, 0.560 ± 0.02, 0.602 ± 0.12, and 0.693 ± 0.07, respectively.

Furthermore, our extracts were richer in total polyphenols and total flavonoids than those evaluated by Martins et al. (2018) [54]. As previously mentioned, extracts from Gorreana (conventional), Gorreana (Soxhlet), Happy Flora (conventional), and Happy Flora (Soxhlet) exhibited TPCs of 863.1, 833.7, 758.3, and 845.3 mg GAE/g extract, respectively, while extracts from commercial green tea, green tea from capsules, “Hysson” green tea, and “Encosta da Bruma” green tea exhibited TPCs of 416 ± 9.95, 272 ± 2.34, 330 ± 4.68, and 361 ± 3.22 mg GAE/g extract, respectively.

Regarding the flavonoid content, extracts from Gorreana (conventional), Gorreana (Soxhlet), Happy Flora (conventional), and Happy Flora (Soxhlet) exhibited TFCs of 701.5, 547.6, 500.2, and 500.0 mg ECE/g extract, respectively, while extracts from commercial green tea, green tea from capsules, “Hysson” green tea, and “Encosta da Bruma” green tea exhibited TFCs of 148 ± 0.2, 139 ± 9.59, 184 ± 0.64, and 165 ± 7.03 mg ECE/g extract, respectively.

Furthermore, compared with the extract from Castro et al. (2019) [17], our extracts’ values were much higher, as their extract presented an AAC of 379.24 ± 10.43 mg GAE/g of extract and a TFC of 119.81 ± 11.70 mg ECE/g of extract.

Finally, after a global analysis of the previously mentioned tests, Gorreana green tea extract was chosen to be incorporated in the whey protein film. Theoretically, the Chá Camélia extract (leaves) would also be an excellent option because of the tea’s high retention potential and sensory characteristics. Additionally, solid–liquid extraction with the Soxhlet apparatus was selected as the extraction method, since it presented a higher yield (43%) than conventional solid–liquid extraction (5.6%).

### 3.2. Antioxidant and Antimicrobial Properties of the Active Film

After the films were developed, their antioxidant properties and antimicrobial properties were evaluated. The films obtained showed good mechanical properties. The active films were characterized by an olive-green hue and a light green tea aroma. Additionally, there was no solubilization between the films and the fresh cheeses.

#### 3.2.1. Evaluation of the Antioxidant Capacity of the Whey Protein-Based Films

After producing the active films, migration tests were carried out, and their antioxidant capacity was evaluated.

**Figure 10:**
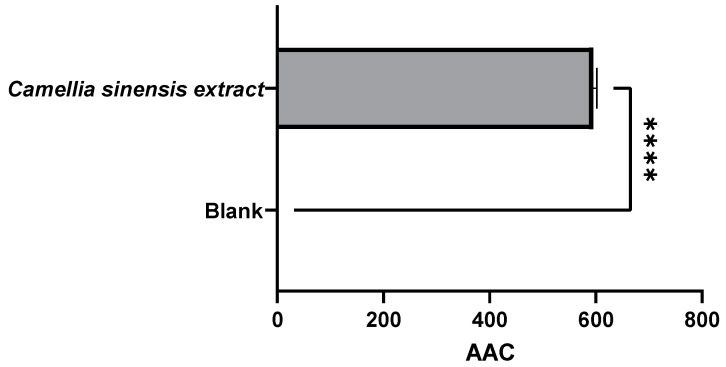
Results of the antioxidant capacity of a β-carotene bleaching inhibition test on the active films after a migration test at 40 °C for 10 days (active film—*C. sinensis* extract vs. control—blank film). β-carotene bleaching inhibition test results are expressed as AAC. **** = *p* ≤ 0.0001.

**Figure 11:**
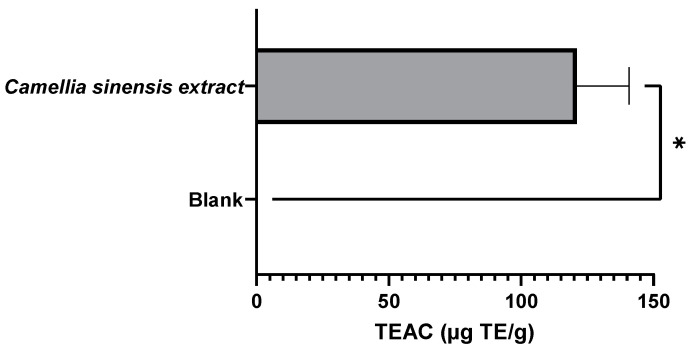
Results of the antioxidant capacity of the active films after a migration test at 40 °C for 10 days (active film—*C. sinensis* extract vs. control—blank film). DPPH radical inhibition test results are expressed in mg Trolox equivalent per g of film (µg TE/g film). * = *p* ≤ 0.05.

As expected, the active film incorporated with 2.5% green tea extract from the Gorreana plantation exhibited a higher antioxidant capacity than the blank film (no added antioxidant extract). As shown in Figure 10 and Figure 11, the film incorporated with tea had not only a high capacity to inhibit lipid peroxidation (≈595.4 AAC) but a high capacity to capture DPPH● free radicals (≈121 µg TE/g). However, compared with the results obtained in the evaluation of the extract of Gorreana obtained by Soxhlet extraction, there were reductions of 43% and 11% in the results for the *β*-carotene bleaching and DPPH radical tests, respectively.

**Figure 12:**
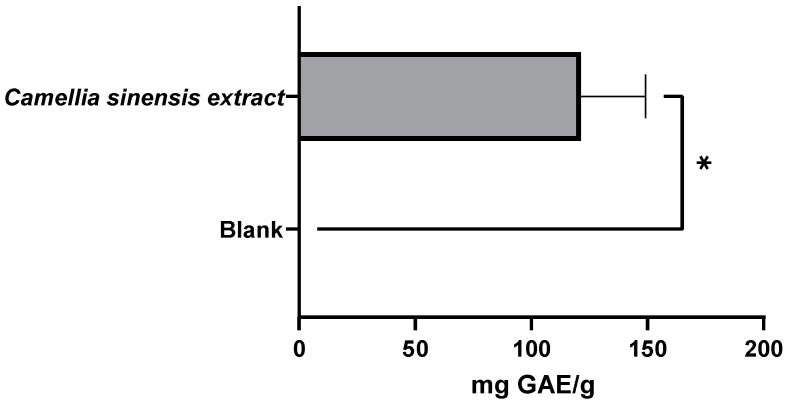
Results of the total phenolic content of the active films after a migration test at 40 °C for 10 days (active film—*C. sinensis* extract vs. control—blank film), expressed in mg gallic acid equivalent per g of film (mg GAE/g film). * = *p* ≤ 0.05.

**Figure 13:**
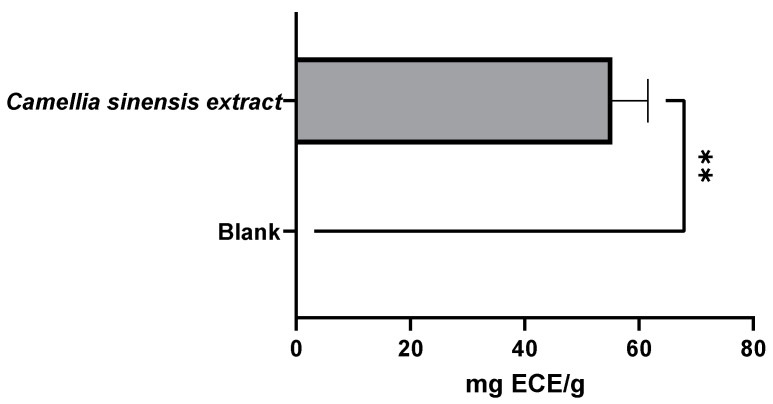
Results of the total flavonoid content of the active films after a migration test at 40 °C for 10 days (active film—*C. sinensis* extract vs. control—blank film), expressed in mg epicatechin equivalent per g of film (mg ECE/g). ** = *p* ≤ 0.01.

After a migration test carried out using ethanol 95% (*v*/*v*) as a food simulant (simulant of fatty foods), the new active film presented a TPC of 121.3 mg GAE/g and a TFC of 55.4 mg ECE/g (Figure 12 and Figure 13). However, compared with the extract of Gorreana obtained by Soxhlet extraction, there were decreases of 93.3% and 76% in TPC and TFC, respectively.

Besides films prepared with green tea extract, there were films prepared with green tea. For these films, the values obtained in the *β*-carotene bleaching and DPPH radical tests were very similar to those obtained during the evaluation of the antioxidant capacity of Gorreana green tea. However, there were reductions of 71.7% and 57.4% in the content of phenolic compounds and flavonoids, respectively. However, it should be noted that the active films developed from the incorporation of green tea did not demonstrate any antioxidant capacity.

According to the literature, very few studies have evaluated the antioxidant capacity of whey protein-based films incorporated with green tea extracts. Pluta-Kubica et al. (2021) [16] and Chalob and Abdul-Rahman (2018) [55] developed edible films using whey protein isolates as biopolymeric matrices and green tea extracts as antioxidant agents. However, the first study used both Furcellaran and whey protein isolate as a biopolymeric matrix, and therefore, it was not possible to compare accurately the antioxidant capacity obtained therein with that obtained here, since we used only whey protein concentrate as a biopolymeric matrix. The second study developed a whey protein isolate-based film and evaluated its antioxidant capacity using a procedure in which the film was cut into aliquots, dissolved in deionized water, and centrifuged. A portion of the solution was mixed with a methanolic solution of DPPH, vortexed, and then incubated. Finally, the solution was centrifuged, and the absorbance was measured. Unfortunately, this procedure was different from that used in this assay to evaluate the antioxidant capacity and therefore does not allow comparison.

In addition, it is important to remark that both studies used whey protein isolate in the formulation of the edible film, and in this study, we used whey protein concentrate, which may influence the film’s antioxidant capacity. As far as we know, according to the available bibliography, no studies have fully compared the antioxidant activity of WPI and WPC films.

Moreover, this research assessed antioxidant capacity only through DPPH scavenging assay, and this parameter does not provide meaningful information about the film’s actual antioxidant capacity when considered alone [33].

#### 3.2.2. Evaluation of the Lipid Oxidation Status of Packaged Goat and Mixture Fresh Cheeses

**Figure 14:**
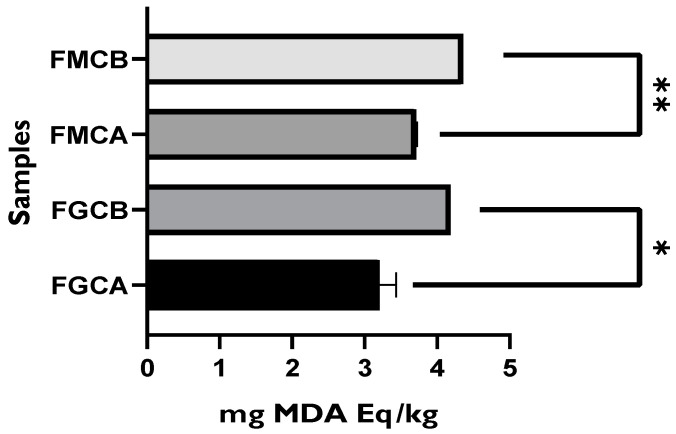
Results of TBARS assay for goat and mixture fresh cheese samples packaged with the control film (without green tea extract) and active film (with green tea extract). FMCB—mixture fresh cheese packaged with the control film; FMCA—mixture fresh cheese packaged with the active film; FGCB—goat fresh cheese packaged with the control film; FGCA—goat fresh cheese packaged with the active film. * = *p* ≤ 0.05, ** = *p* ≤ 0.01.

The TBARS (thiobarbituric acid reactive substances) assay detects lipid peroxidation through measuring malondialdehyde (CH_2_(CHO)_2_) (MDA) [17]. For this assay, a calibration curve (y = 17.657x − 0.0326; r^2^ = 0.9905) was built by plotting different concentrations of 1,1,3,3-tetramethoxypropane (10–55 µg/mL) to express the results in mg of malonaldehyde equivalent per kg of sample (mg MDA Eq/kg). Figure 14 demonstrates that all the fresh cheeses packaged with the whey protein-based active film (incorporated with green tea extract) presented lower MDA content than those packaged with the control film (without green tea extract). Goat and mixture fresh cheeses packaged with the active film exhibited 3.2 and 3.7 mg MDA/kg sample, respectively, while those packaged with the control film exhibited 4.2 and 4.4 mg MDA/kg sample, respectively. In addition, within the same type of cheese, significant differences were found between the cheeses wrapped with the active film and those wrapped with the control film. Furthermore, none of the fresh cheese samples packaged with the active and control films presented MDA values above 0.5 mg MDA/kg sample, which is the threshold in which off flavors are perceived [8]. However, in a previous analysis, after 6 days of storage, unpackaged mixture fresh mixture cheese presented 5.2 mg MDA/kg sample. These results suggest that, within a week, these active packages have the potential to inhibit fresh cheese lipid oxidation. Nevertheless, it is important to remark that TBARS has some limitations, since it depends on substances reactive to thiobarbituric acid, which degrade along with lipid oxidation.

Andrade et al. [8] and Castro et al. [17], through the TBARS assay, evaluated the oxidation status of salami and smoked salmon, respectively, both packaged with an active film similar to that used in this study. Andrade et al.’s results showed that after one week, salami packaged with the active film presented approximately 12.5% less MDA content when compared to the sample packaged with the control film. Castro et al. [17] demonstrated that after one week, smoked salmon packaged with the active film had approximately 24% less MDA content than the control. Comparing the present study’s results with those of the previously mentioned studies, our active package had efficient performance, as within one week, goat and mixture fresh cheese packaged with the active film had 24% and 15% less MDA content, respectively, than the corresponding fresh cheeses packaged with the control film. It is important to remark, however, that the food matrices were different and that fresh cheese is much more perishable than salami or salmon, which explains the higher levels of oxidation of fresh cheese.

#### 3.2.3. Microbiological Analysis of Packaged Fresh Cheeses

In this study, the total count of microorganisms at 30 °C, *E. coli*, and coagulase-positive *staphylococci* were investigated, since these are indicators not only of food quality but of compliance within the scope of good hygiene practices during cheese production and distribution. Additionally, since fresh cheeses are usually stored at refrigerated temperatures (5 °C), a psychrophile count was carried out. The counts of these different microorganisms were carried out on goat fresh cheese and mixture (sheep and goat) fresh cheese produced on the day. Furthermore, microorganism counts on the two types of fresh cheeses wrapped with active films (active) and control films (without green tea extract—blank) were carried out after one week of storage at 5 °C.

**Figure 15:**
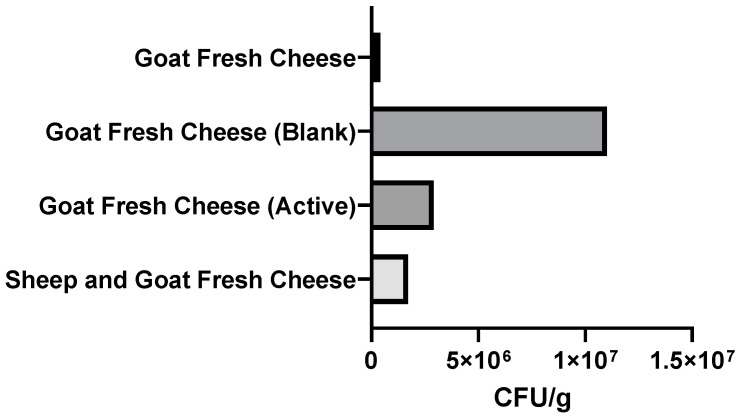
Results of total microorganism count at 30 °C, expressed as colony-forming units per g of fresh cheese (CFU/g fresh cheese). Total microorganism counts were carried out on goat fresh cheese (Goat Fresh Cheese) and mixture fresh cheese (Sheep and Goat Fresh Cheese) produced on the day. Furthermore, both cheeses were wrapped with the edible active film (Goat Fresh Cheese (Active) and Sheep and Goat Fresh Cheese (Active)) and with the control film (Goat Fresh Cheese (Blank) and Sheep and Goat Fresh Cheese (Blank)) and stored at 5 °C in order to simulate standard storage conditions. After one week of storage at refrigerated temperatures, a total microorganism count was carried out. The numbers of colonies on Goat Fresh Cheese (Active) and blank were uncountable and are therefore not shown.

In terms of food quality, it can be concluded from Figure 15 that the active films effectively inhibited the growth of mesophilic microorganisms, and the goat cheese coated with the active film presented a significantly lower count of CFU/g (2.9 × 10^6^ CFU/g) than the blank coated cheese (1.1 × 10^7^ CFU/g). In mixture fresh cheese (goat and sheep), the colonies were countless; however, to the naked eye, the active film-coated sample appeared to have fewer colonies compared to the blank coated sample (fresh cheese coated with control film).

**Figure 16:**
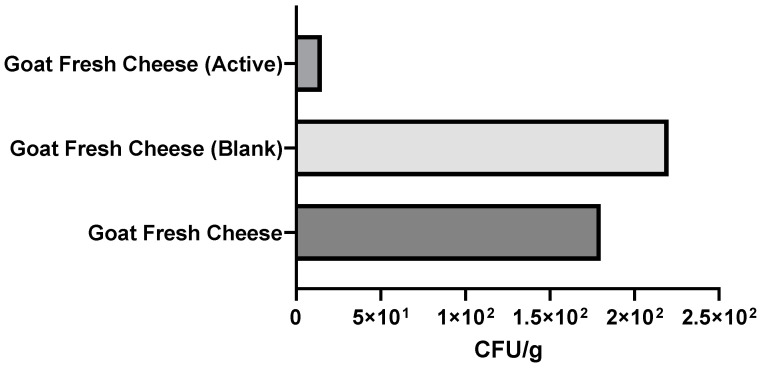
Results of the *Escherichia coli* colony count in goat fresh cheese, expressed as CFU per g of fresh cheese (CFU/g fresh cheese). An *Escherichia coli* colony count was carried out on goat fresh cheese produced on the day (Goat Fresh Cheese). Furthermore, the cheese was wrapped with the edible active film (Goat Fresh Cheese (Active)) and with the control film (Goat Fresh Cheese (Blank)) and stored at 5 °C in order to simulate standard storage conditions. After one week of storage at refrigerated temperatures, an *Escherichia coli* colony count was carried out.

**Figure 17:**
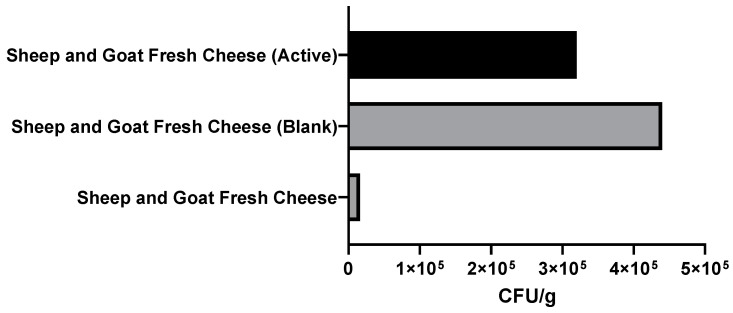
Results of the *Escherichia coli* colony count in mixture (sheep and goat) fresh cheese, expressed as CFU per g of fresh cheese (CFU/g fresh cheese). An *Escherichia coli* colony count was carried out on mixture fresh cheese produced on the day (Sheep and Goat Fresh Cheese). Furthermore, the cheese was wrapped with the edible active film (Sheep and Goat Fresh Cheese (Active)) and with the control film (Sheep and Goat Fresh Cheese (Blank)) and stored at 5 °C in order to simulate standard storage conditions. After one week of storage at refrigerated temperatures, an *Escherichia coli* colony count was carried out.

In the context of food safety, considering that despite working as a hygiene indicator, some strains of *E. coli* are pathogenic, Figure 16 and Figure 17 show inhibition of the growth of microorganisms in the samples coated by the active film. In fact, in goat cheese (Figure 16), there was even a significant reduction in the number of colonies (1.5 × 10 CFU/g) when compared with fresh cheese produced on the day (1.8 × 10^2^ CFU/g) and, of course, with the blank coated cheese (2.2 × 10^2^ CFU/g).

In the mixture cheese (Figure 17), there was no decrease in the number of colonies when compared with fresh cheese produced on the day (1.6 × 10^4^ CFU/g); however, the cheese coated by the active film had a lower number of CFU/g (3.2 × 10^5^ CFU/g) than the blank-coated cheese (4.4 × 10^5^ CFU/g). Note that neither coagulase + *staphylococci* nor *psychrophiles* grew in any of the samples. At least six edible films have been developed to date for unripened cheeses of which the antimicrobial activity has been assessed. Pluta-Kubica et al. (2020) [14] developed active edible furcellaran/whey protein films with yerba mate and white tea extracts and applied them to fresh soft rennet-curd cheese. The microbiological quality of the cheese was determined through total count of bacteria (TBC), yeast, molds, and coliforms. In 2021, Pluta-Kubica et al. [16] published another article in which they applied furcellaran/whey protein isolate films containing extracts (Pu-erh or green tea) to an acid-curd cheese. This study determined Lactococcus, TBC, yeast, molds, and coliforms. Furthermore, Santacruz and Castro (2018) [28] developed a cassava starch edible coating incorporated with *Lactobacillus acidophilus* and applied it to Manaba fresh white cheese. The antibacterial activity of L. acidophilus was assessed against a *Salmonella* strain previously isolated from Manaba cheese. The mesophilic aerobic bacteria on the cheese were also determined. Moreover, Di Pierro et al. (2011) [15] developed a chitosan/whey protein coating to extend ricotta cheese’s shelf life and determined its antimicrobial activity against aerobic, mesophilic, and psychotropic microorganisms. In addition, Mileriene et al. (2021) [13] evaluated the effect of liquid whey protein concentrate-based edible coating enriched with cinnamon carbon dioxide extract on eastern European curd cheese’s quality and shelf life. To assess the microbiological quality, they enumerated TBC, lactic acid bacteria, *Staphylococcus* spp., Enterobacteria, coliforms, yeast, and molds. Finally, Jutinico-Shubach et al. [29] evaluated the antilisterial activity of chitosan-based edible coating incorporating cell-free supernatant from *Pediococcus pentosaceus* 147 on the preservation of Colombian fresh cheese and performed microbiological analysis by determination of *L. monocytogenes*, mesophilic bacteria, psychrophilic bacteria, and total mold and yeast counts.

Most of the aforementioned research involved developing attractive edible films/coatings to apply to unripened cheese surfaces. However, the microbiological quality of the various unripened cheeses was evaluated through a wide variety of parameters, not allowing adequate comparisons. In addition, it is essential to remark that the microbiological quality varies between unripened cheeses, as some require starter cultures (preparations of living microorganisms, which are deliberately added to foods to take advantage of the compounds or products derived from their metabolism or enzymatic activity) to be produced [56]. Nevertheless, some authors have evaluated the microbiological quality of cheeses through enumeration of mesophilic bacteria, allowing comparisons with the present study results.

In a study by Pluta-Kubica et al. (2020) [14], within one week, the TBC of uncoated fresh soft rennet-curd cheese diminished from 8.8 log CFU/g to 8.6 log CFU/g. In addition, after one week, cheese coated with furcellaran/whey protein isolate exhibited a TBC of 8.8 log CFU/g, cheese coated with furcellaran/whey protein isolate incorporated with yerba mate extract exhibited a TBC of 8.6 log CFU/g, and cheese coated with furcellaran/whey protein isolate incorporated with white tea extracts exhibited a TBC of 8.5 log CFU/g. Even though differences between the active films and the control were minimal or nonexistent, the authors were able to obtain good results in the scope of their research because TBC, in fresh soft rennet-curd cheese, includes primarily lactic acid bacteria, which are not spoilage microorganisms. In 2021, Pluta-Kubica et al. [16] demonstrated that within one week, the TBC of uncoated fresh acid-curd cheese diminished from 7.5 log CFU/g to 6.5 log CFU/g. In addition, after one week, cheese coated with furcellaran/whey protein isolate exhibited a TBC of 5.9 log CFU/g, cheese coated with furcellaran/whey protein isolate incorporated with green tea extract exhibited a TBC of 5.9 log CFU/g, and cheese coated with furcellaran/whey protein isolate incorporated with Pu-erh extracts exhibited a TBC of 6.8 log CFU/g. In this study, however, none of the edible films used improved the microbiological quality of an acid-curd cheese.

Santacruz and Castro (2018) [26] demonstrated that within ten days, mesophilic bacteria on uncoated Manaba cheese increased from 8.60 log CFU/g to 9.20 log CFU/g. After ten days, Manaba cheese coated with cassava starch film containing free *L. acidophilus* presented 4.10 log CFU/g, while cassava starch film containing encapsulated L. acidophilus presented 5.90 log CFU/g. Moreover, according to Di Pierro et al. (2011) [15], the viable numbers of mesophilic bacteria on uncoated ricotta cheese after one week increased from ≈6.5 log CFU/g to ≈7.0 log CFU/g, while on the coated ricotta cheese, the viable numbers of mesophilic bacteria were maintained (≈6.5 log CFU/g). Finally, in a 2021 study by Mileriene et al. [13], the TBC diminished from 7 to 4 log CFU/g in all samples during storage (31 days). However, after ten days of storage, the curd cheese TBC increased from 6.91 log CFU/g to 7.15 log CFU/g, and the coated curd cheese TBC increased from 6.92 log CFU/g to 7.26 log CFU/g. On the other hand, vacuum-packed curd cheese’s and coated and vacuum-packed curd cheese’s TBCs diminished from 6.91 log CFU/g to 6.39 log CFU/g and from 6.90 log CFU/g to 6.47 log CFU/g, respectively.

Regarding the viable numbers of mesophilic bacteria, converting CFU/g to log CFU/g, after one week, our uncoated goat cheese presented ≈7.0 log CFU/g, and our coated goat cheese presented ≈6.5 log CFU/g. In addition, it is important to recall that Latin-style fresh cheese manufacture does not require starter cultures. This actively demonstrates that our whey protein-based edible film incorporated with green tea extract had good performance compared with the formerly developed ones. However, this parameter alone does not allow any conclusions.

## 4. Conclusions

In the present study, a screening of the antioxidant capacity of Portuguese and Asian green tea infusions was successfully performed. The green tea provided by Chá Camélia was characterized for the first time, as was the infusion prepared with flowers of *C. sinensis*. The former revealed a great antioxidant capacity and outstanding potential to retain antioxidant capacity. Moreover, extracts from two brands (a Portuguese and an Asian brand) of green tea were prepared, and their yields and antioxidant capacities were evaluated, demonstrating their high antioxidant potential. It is important to remark that in future studies, qualitative analysis of the extracts should be performed. Because of limitations in the samples’ availability, Chá Camélia samples were not used to prepare extracts. However, in the future, these samples should be considered. In addition, a comparison between conventional solid–liquid extraction and Soxhlet extraction suggested that the former may be the most adequate because of its yield. Furthermore, the use of the Soxhlet apparatus does not allow stirring and may lead to the thermal degradation of compounds, since this process exposes the samples to high temperatures for a long time [57].

Additionally, active whey protein-based films incorporated with green tea extracts (2.5%) were successfully developed, and their application as a packaging material for Latin-style fresh cheeses effectively preserved this product.

Evaluation of the antioxidant capacity of the developed films was also carried out and indicated that whey protein-based films incorporated with green tea extracts might successfully prevent or inhibit lipid oxidation of fresh cheeses. In addition, through the TBARS assay, the capacity of the new active film to delay lipid oxidation of fresh cheese during storage was confirmed.

Tested over a week, the cheese samples wrapped with active edible films were characterized by lower counts of mesophilic bacteria and *E. coli*. However, the active edible films altered the color and hardness of fresh cheeses. Further studies are required to fully assess whether this package can extend Latin-style fresh cheese’s shelf life and to evaluate the impact of the active films developed on the organoleptic quality of Latin-style fresh cheese samples. In future studies, the total microorganism count at 30 °C should be repeated for mixture fresh cheese wrapped with control and active films in order to obtain more results. Additional studies should be performed to fully evaluate the antimicrobial capacity concerning foodborne bacteria such as *Listeria monocytogenes*.

The developed whey protein-based films incorporated with green tea extracts are disruptive packaging materials that have been successfully used to preserve Latin-style fresh cheese. Furthermore, this packaging is environmentally friendly, as it is biodegradable and renewable and therefore generates zero waste. Moreover, its manufacture adds value to whey, making it more profitable to companies. However, greater understanding of the subject and sensory analysis are required to better understand the magnitude of the cost/benefit ratio and whether or not this innovation would be accepted by consumers.

## Figures and Tables

**Table 1:** Nutrition declaration per 100 g of goat fresh cheese and mixture fresh cheese samples.

Nutrition Declaration per 100 g of Product (Queijaria Licínia, Rabaçal, Portugal)	Goat Fresh Cheese	Mixture Fresh Cheese
Energy (kJ)–Energy (kcal)	10.39–259	10.82–261
Fat (g) Of which saturated fatty acids (g)	18.8	20.8
	12.98	13.93
Carbohydrates Of which sugars	4.2	4.6
	0.5	0.9
Protein (g)	16.6	13.8
Salt (g)	1.10	0.55
Water content (g)	45.82	45.42

## Data Availability

Data is contained within the article.
